# Supplementary material for: Dipole-field-assisted charge extraction in metal-perovskite-metal back-contact solar cells
Source: Nat Commun. 2017 Sep 20;8:613. doi: 10.1038/s41467-017-00588-3 (PMC5606993; doi:10.1038/s41467-017-00588-3)
Supplement: Supplementary file 1 — Supplementary Information [file 41467_2017_588_MOESM1_ESM.pdf]

Description of Supplementary Files

File Name: Supplementary Information
Description: Supplementary Figures

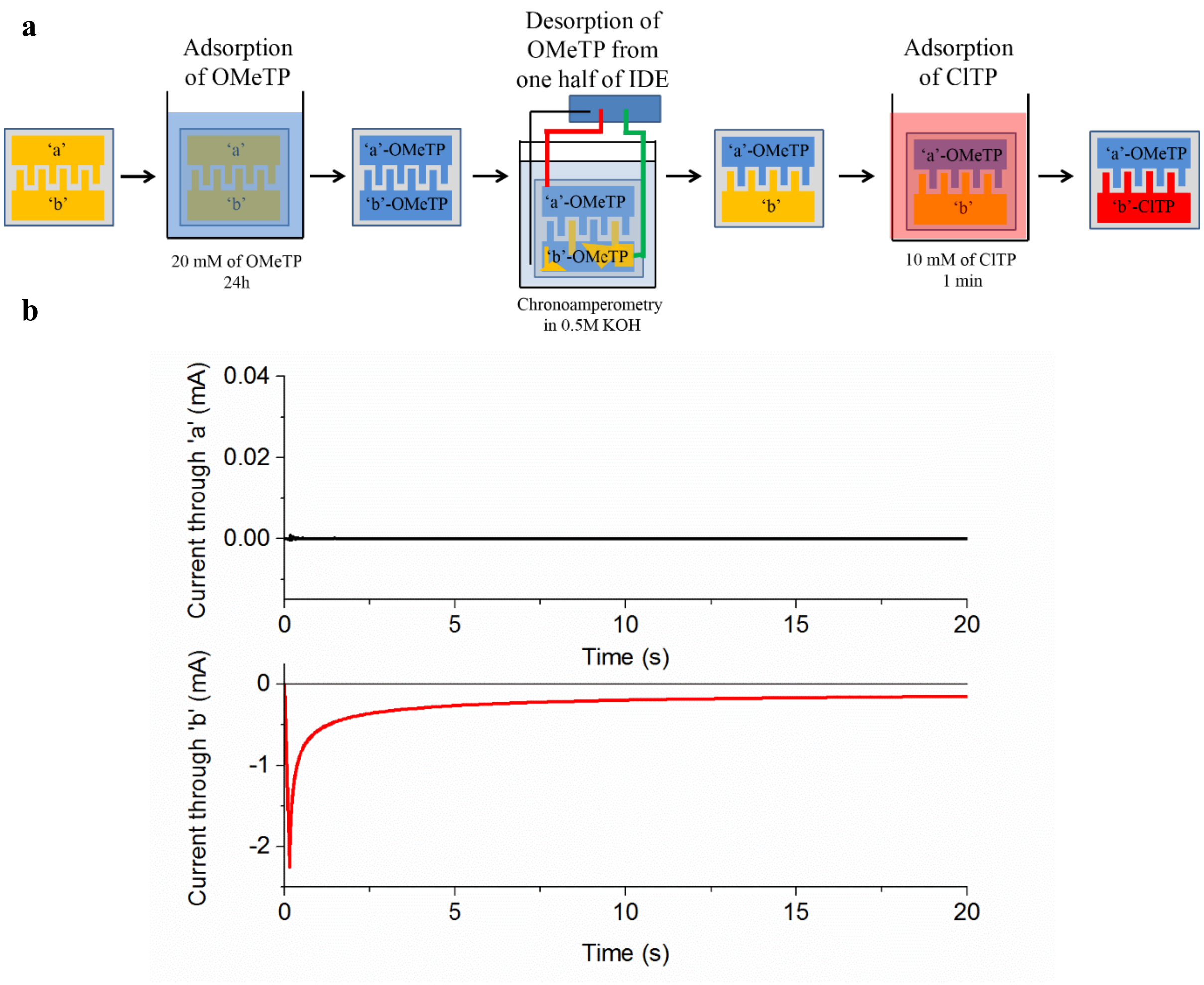

Supplementary Figure 1 | IDA modification. (**a**) Stepwise protocol for thiol modification of gold IDA. (**b**) Simultaneous chronoamperometry monitored on the modified contacts 'a' (black) and 'b' (red) in a 0.5M KOH aqueous solution.

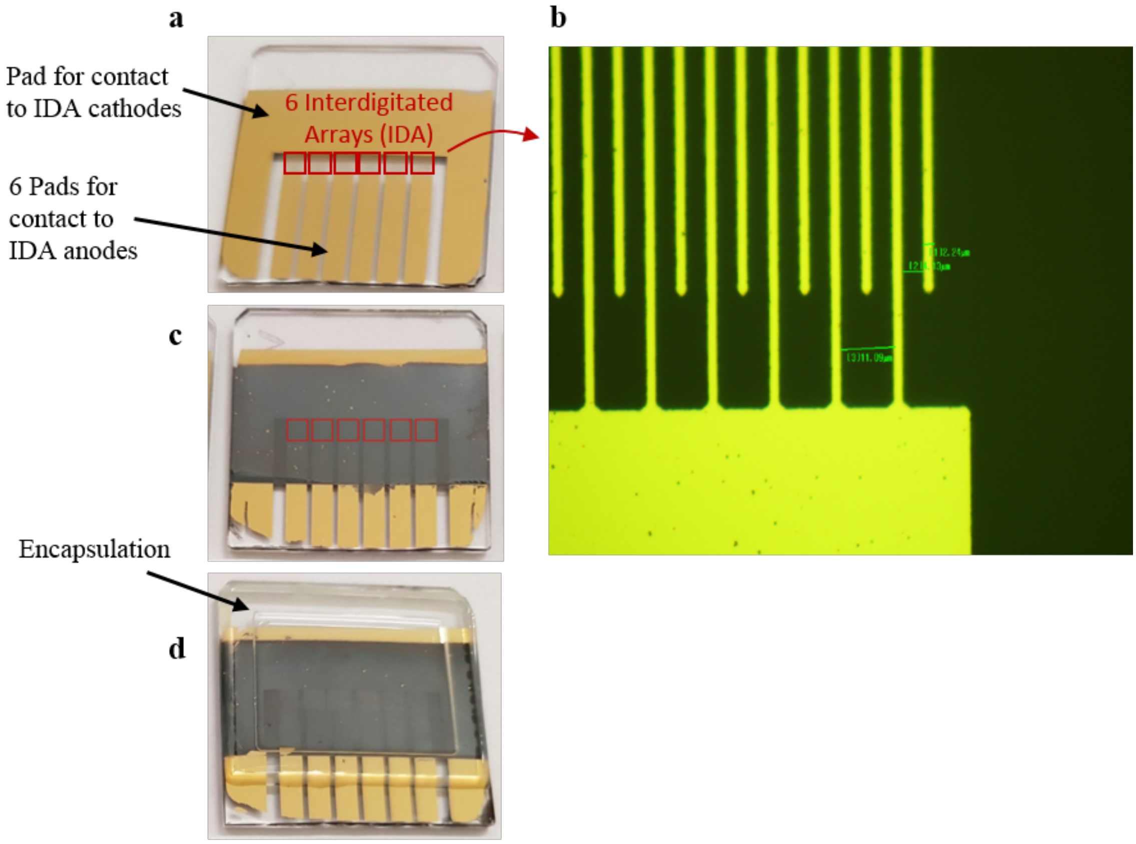

Supplementary Figure 2 | Photographic images of IDA and bc-PSC. (**a**) Gold patterned glass substrate with 6 IDA (each 2 mm × 2 mm) indicated by the 6 red squares. It also consists of larger gold pads to enable electrical connections to be made. (**b**) An optical image showing the IDA in more detail. The gap between electrodes is about 4.3 µm and the electrode width is about 2.2 µm. (**c**) After perovskite deposition to form the bc-PSC. (**d**) Bc-PSC with encapsulation.

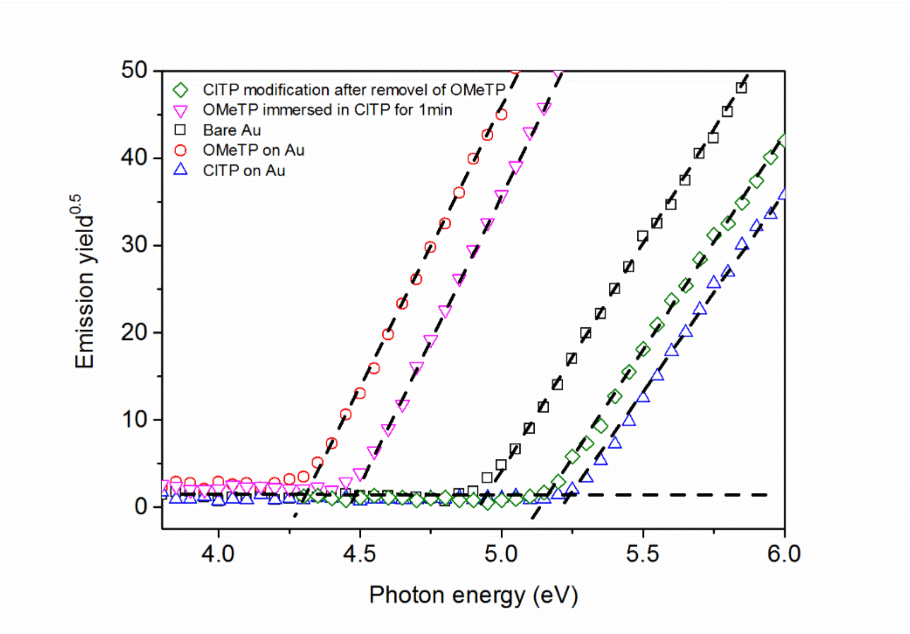

Supplementary Figure 3 | PESA spectra. PESA spectra of gold films with and without SAM-modification. PESA results show that bare or pristine gold has a work function of about 4.93 eV. After SAM-modification with OMeTP and ClTP, the work function of gold shifted to 4.35 eV and 5.25 eV, respectively. In order to simulate the same SAM-modification as with IDAs, gold with a OMeTP monolayer was immersed into a ClTP in ethanol solution for 1 min, with the resulting work function shifting to 4.51 eV due to thiol exchange. In order to simulate the SAM-modification for the second set of IDA fingers, a OMeTP monolayer on gold film was electrochemically removed from the surface and the same substrate was immersed into a ClTP in ethanol solution, with its work function recorded to be 5.16 eV.

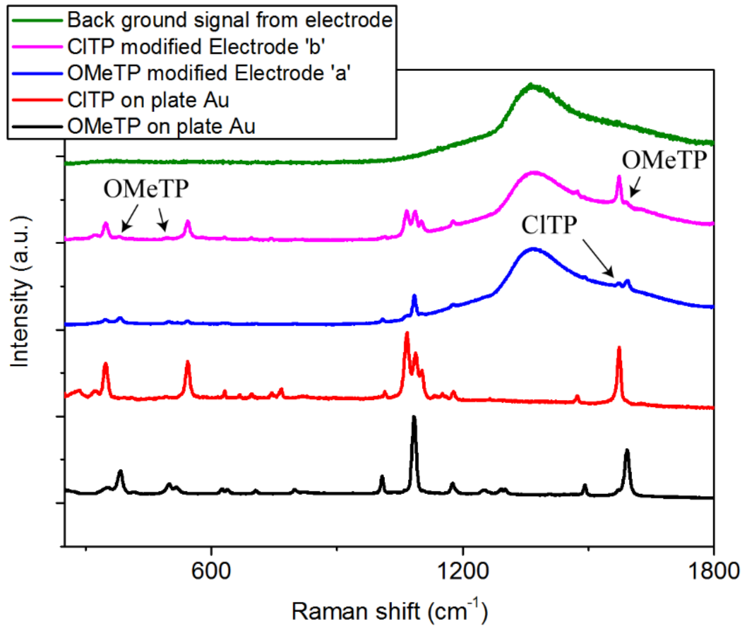

Supplementary Figure 4 | Raman spectra. Raman spectra of OMeTP on plate gold (black), ClTP on plate gold (red) and on SAMs modified IDA (electrode ‘a’ in blue, electrode ‘b’ in magenta and background in green). ClTP peak on electrode ‘a’ and OMeTP peak on electrode ‘b’ is identified and labeled.

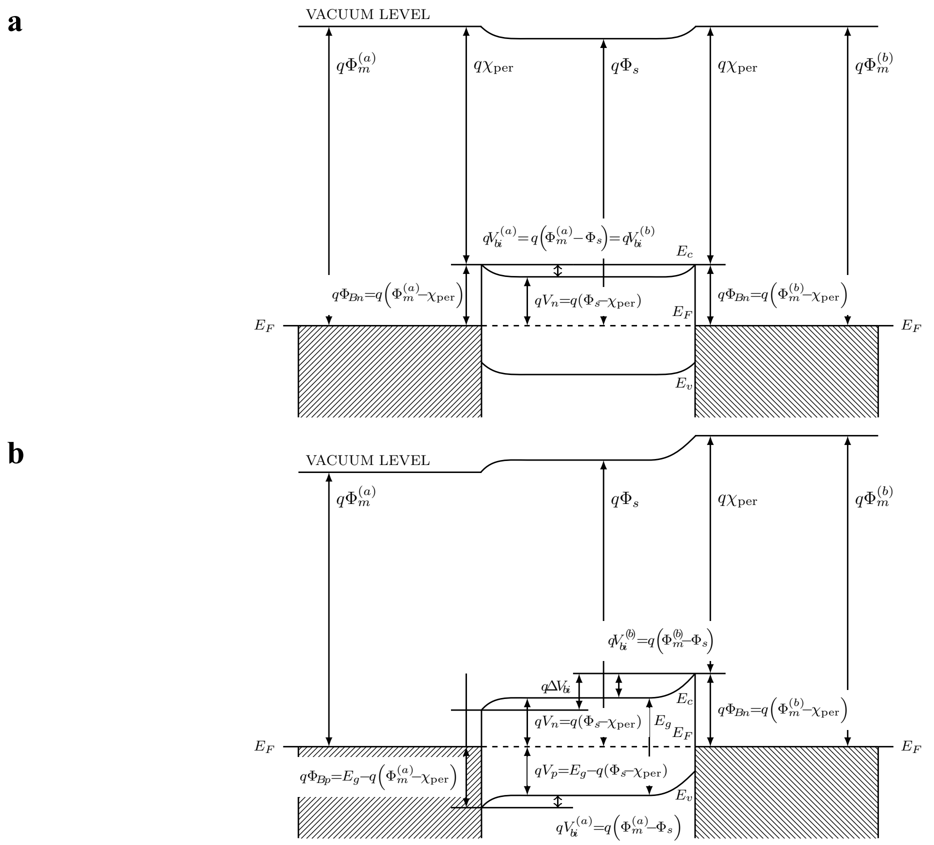

Supplementary Figure 5 | Band diagrams. Thermal equilibrium band diagram of (**a**) Au-MAPbI$_3$-Au band structure (without poling) and (**b**) 'a'-MAPbI$_3$-'b' structure ('a' refers to electrode modified with OMeTP and 'b' refers to electrode modified with ClTP) where E_F - Fermi level, E_c and E_v are conduction and valence band edges for MAPbI$_3$, respectively, $q\Phi_m^{(a)}$ and $q\Phi_m^{(b)}$ are the respective work functions of the OMeTP and ClTP modified contacts, $q\Phi_s$ - work function of MAPbI$_3$, $q\chi_{per}$ - electron affinity of MAPbI$_3$, $q\Phi_{Bp}$ and $q\Phi_{Bn}$ are the respective barrier heights at the 'a'-MAPbI$_3$ and MAPbI$_3$-'b' M-S interfaces (in the case of Au-MAPbI$_3$-Au structure $q\Phi_{Bn}$ is barrier height for both, at the Au-MAPbI$_3$ and MAPbI$_3$-Au M-S interfaces), $qV_{bi}^{(a)}$ and $qV_{bi}^{(b)}$ are the built-in potentials at the 'a'-MAPbI$_3$ and MAPbI$_3$-'b' M-S interfaces, respectively, $q\Delta V_{bi}$ is the difference in built-in potential between 'a' and 'b' M-S interfaces, qV_n is a potential difference between Fermi level and E_c, while qV_p is a potential difference between Fermi level and E_v, E_g is the band gap of MAPbI$_3$. Band diagrams for other asymmetric 'a'-MAPbI$_3$-Au, Au-MAPbI$_3$-'b' and symmetric 'a'-MAPbI$_3$-'a' and 'b'-MAPbI$_3$-'b' devices can be constructed based on combination of (**a**) and (**b**) or only (**b**), respectively.

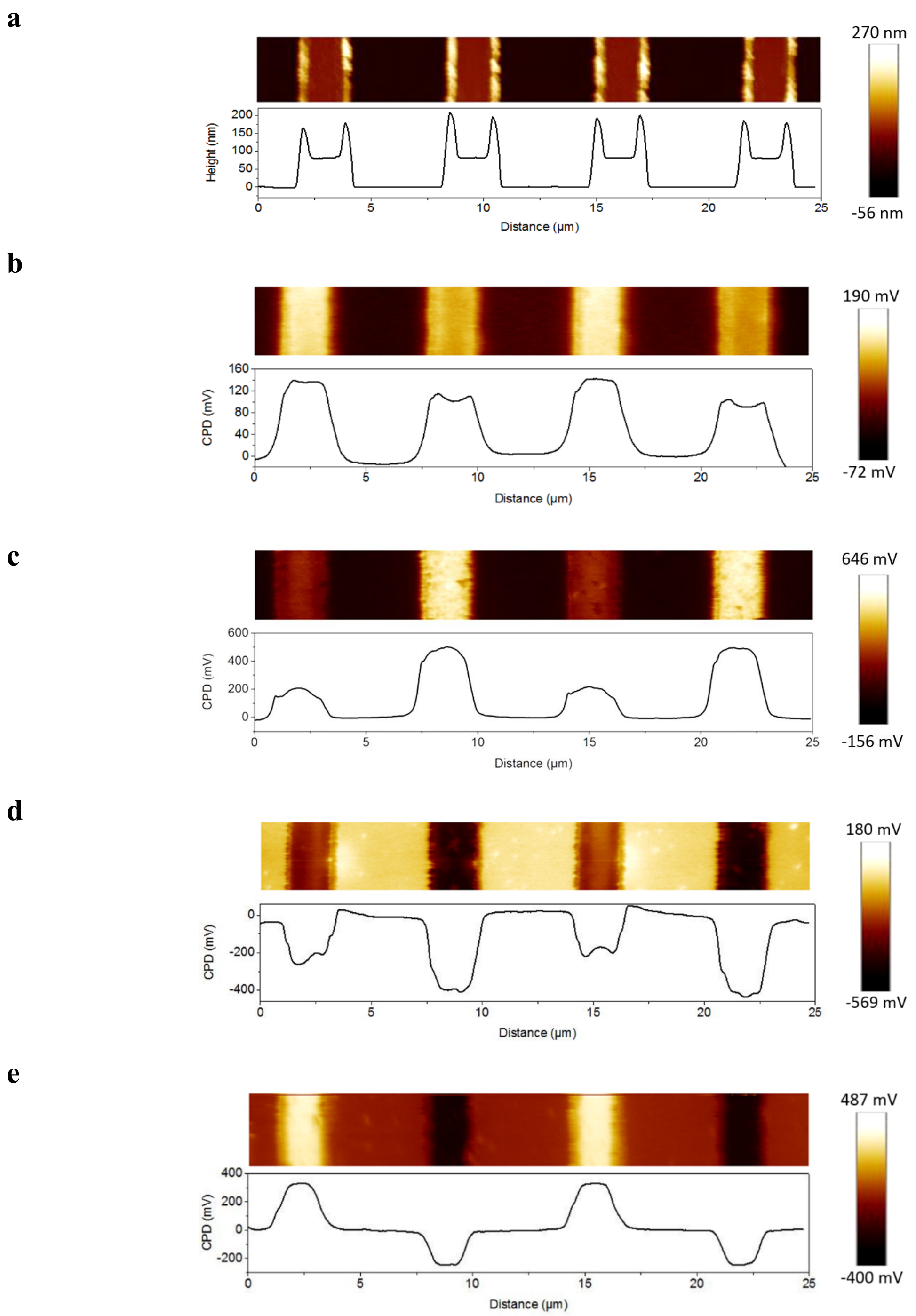

Supplementary Figure 6 | AFM and KPFM images of SAM-modified IDAs. (**a**) Surface topography of gold IDAs. (**b**) Surface potential of bare gold IDAs, which underwent an electrochemical treatment in KOH without the presence of SAMs. (**c**) Surface potential of an IDA modified with OMeTP only and selective desorption treatment from one side. (**d**) Surface potential of an IDA modified with ClTP only and selective desorption treatment from one side. *CPD* difference is around 200 mV. (**e**) Surface potential of OMeTP- and ClTP-modified IDA. *CPD* difference is around 580 mV.

a

b

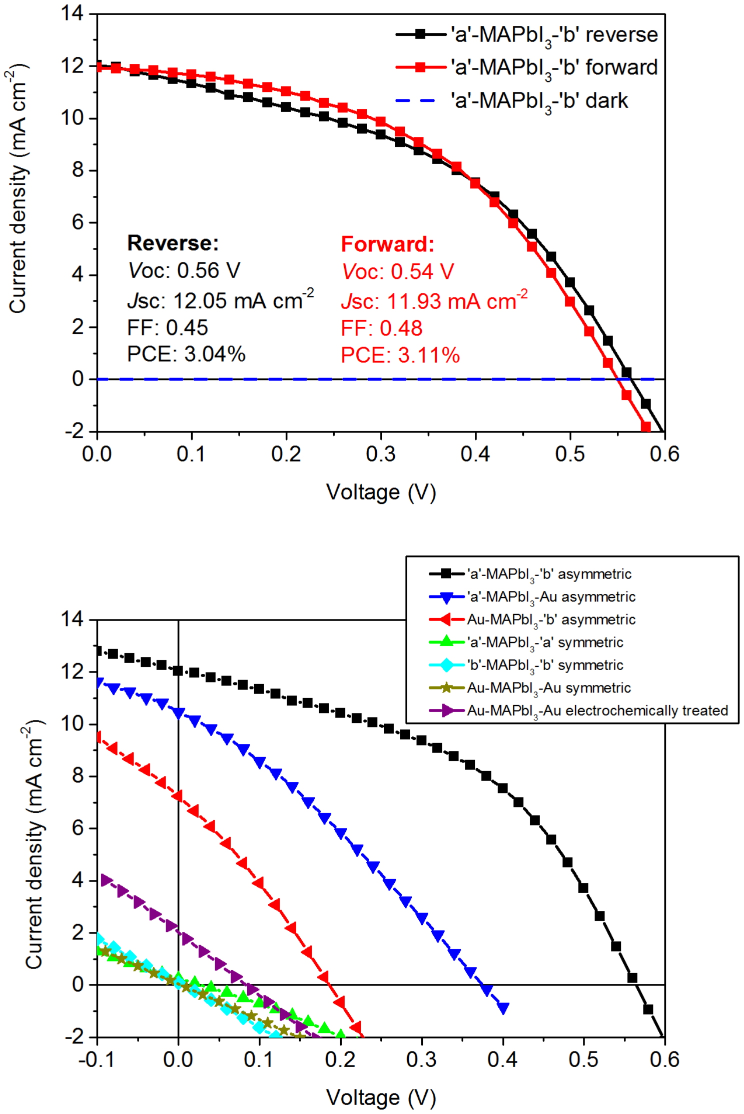

Supplementary Figure 7 | *J-V* characteristics. (**a**) An unencapsulated champion 'a'-$MAPbI_3$-'b' cell ('a' is electrode modified with OMeTP and 'b' is electrode modified with ClTP). (**b**) Comparison of *J-V* characteristics for unencapsulated asymmetric and symmetric devices. *J-V* curves were recorded in reverse scan mode ($V_{OC} \rightarrow 0$). The 'a'-$MAPbI_3$-'b' device's V_{OC}, J_{SC}, *FF* and PCE values are 0.56 V, 12.1 mA cm^{-2}, 45% and 3.04%, respectively. The 'a'-$MAPbI_3$-Au device shows 0.39 V, 10.4 mA cm^{-2} and 31.2% for V_{OC}, J_{SC} and *FF* values, respectively, with a PCE of 1.26%. For the Au-$MAPbI_3$-'b' device V_{OC}, J_{SC}, *FF* and PCE values are 0.2 V, 7.59 mA cm^{-2}, 29% and 0.44%, respectively. Au-$MAPbI_3$-Au electrochemically treated device refers to device fabricated on bare gold IDA which had experienced identical electrochemical treatments as in 'a'-$MAPbI_3$-'b', but without the presence of SAMs. Such a device showed some photovoltaic effect, but compared to devices with proper SAM-modification its photovoltaic effect is minor. The asymmetry in this device can be attributed to trapping of ions or absorption of organic impurities to one half of the IDA during the electrochemical treatment. The KPFM images for such an electrode also showed a minor difference in *CPD* between opposing parts of the IDA (see Supplementary Fig. 7b).

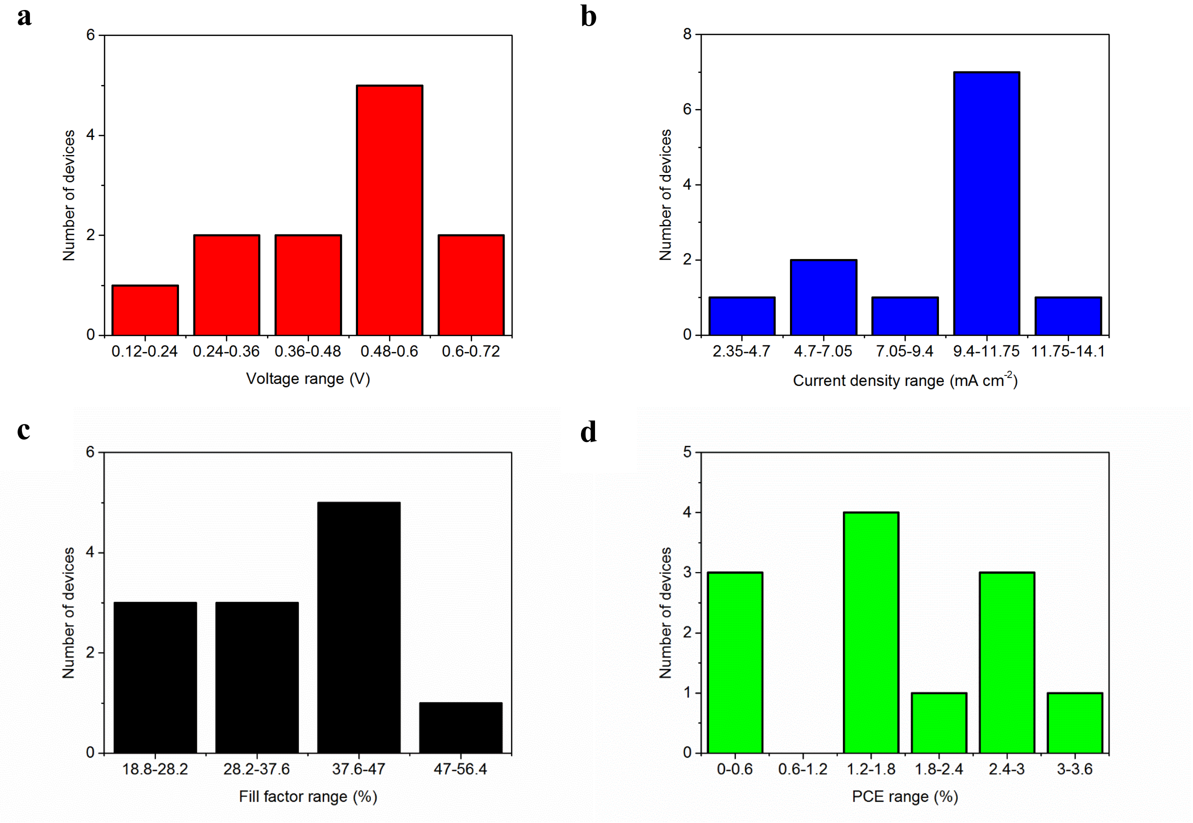

Supplementary Figure 8 | Statistics of solar cell performance from a batch of 12 devices. (**a**) Distribution of open circuit voltage. (**b**) Distribution of current density. (**c**) Distribution of fill factor. (**d**) Distribution of power conversion efficiency.

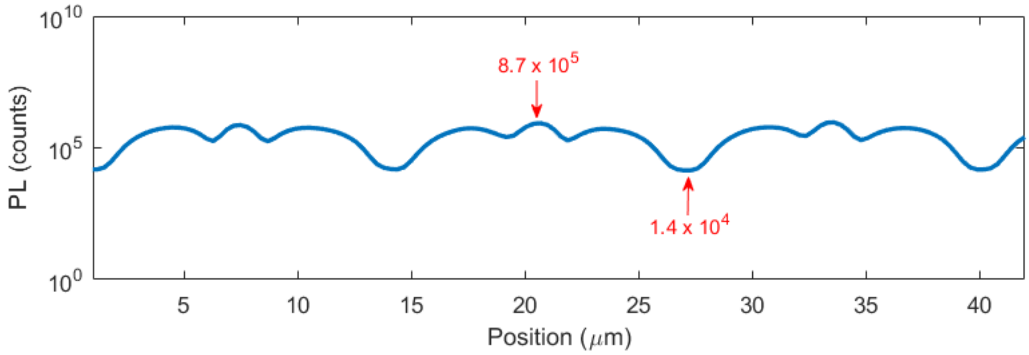

Supplementary Figure 9 | Photoluminescence profile. PL profile data on logarithmic scale. The intensity of the peak and trough differs by 2 orders of magnitude.

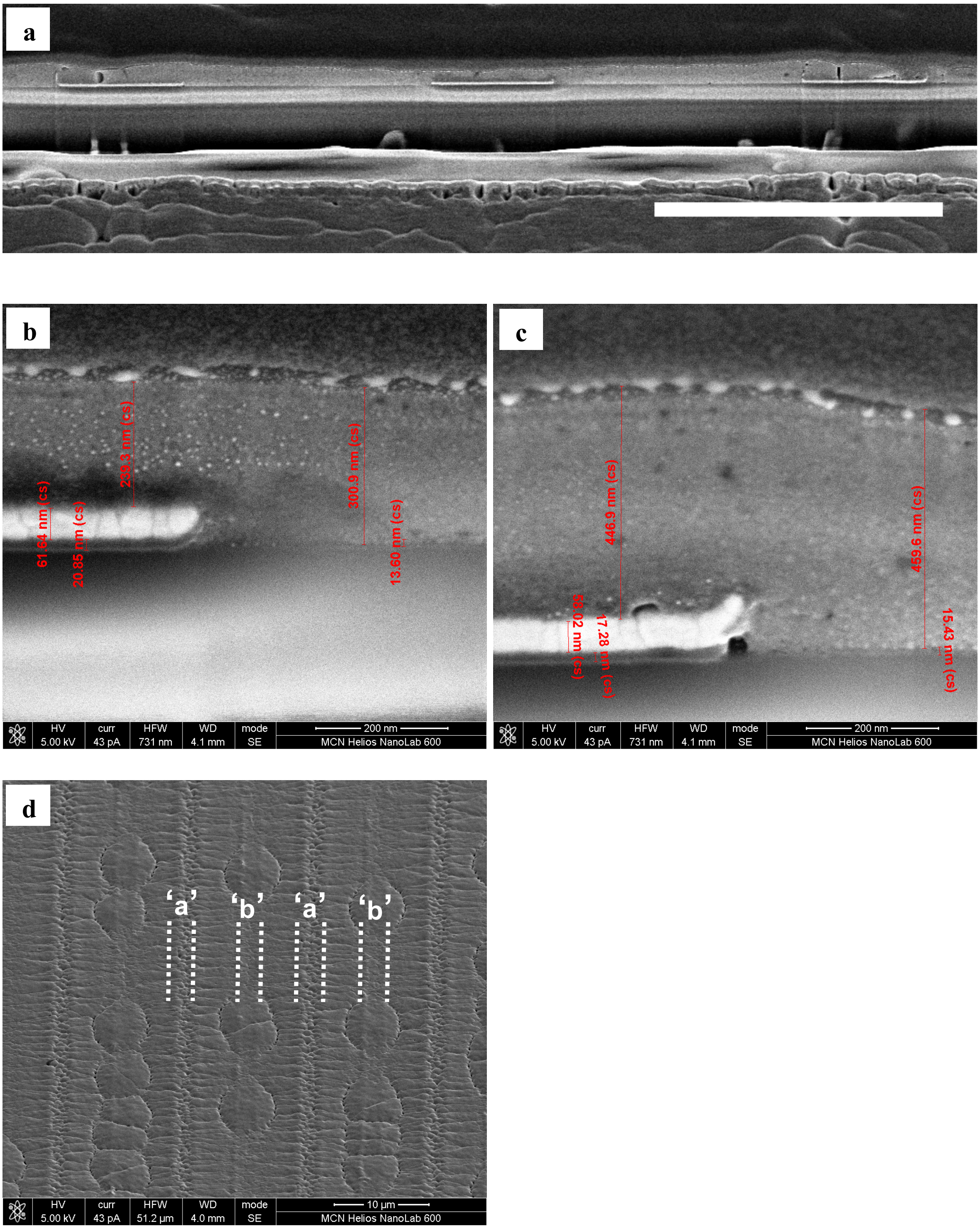

Supplementary Figure 10 | SEM images. (**a**) FIB cross section image of an 'a'-$MAPbI_3$-'b' device ('a' is electrode modified with OMeTP and 'b' is electrode modified with ClTP). Scale bar is 5 µm. (**b**) High magnification cross-section image of OMeTP-modified gold electrode ('a'). (**c**) High magnification cross-section image of ClTP-modified gold electrode ('b'). The difference in $MAPbI_3$ thickness over 'a' and 'b' electrode is ~200 nm. (**d**) SEM top-view of an 'a'-$MAPbI_3$-'b' device. The markers on the image indicate the positions of 'a' and 'b' interdigitated electrodes.

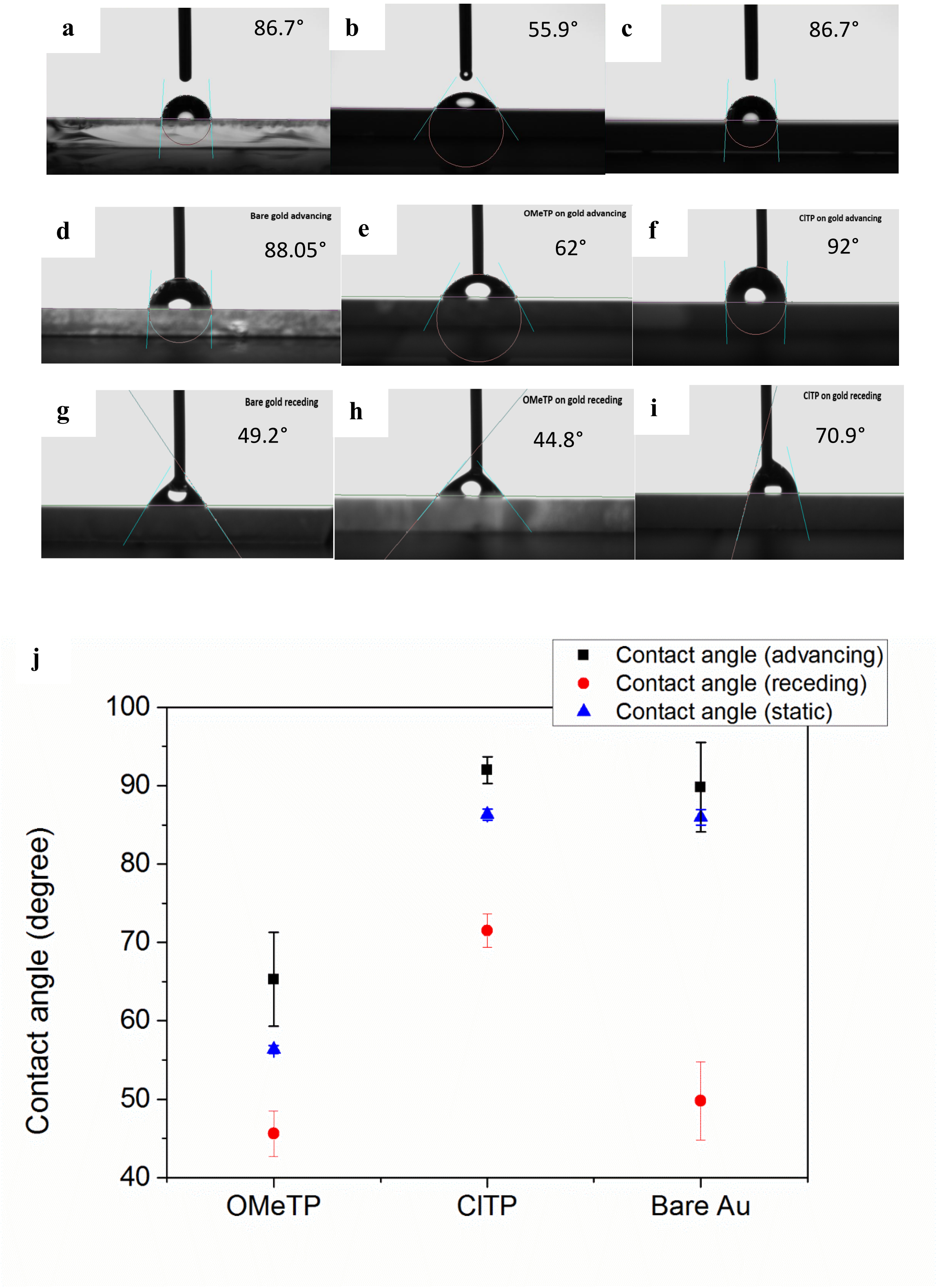

Supplementary Figure 11 | Contact angle measurements. (**a, b**) Static contact angle measurements for ClTP on gold and OMeTP on gold respectively. (**c**) Static contact angle measurements for bare gold. (**d, e, f**) Advancing contact angle measurement for bare gold, OMeTP on gold, and ClTP on gold, respectively. (**g, h, i**) Receding contact angle measurement for bare gold, OMeTP on gold, and ClTP on gold, respectively. (**j**) Summary of contact angle measurements on gold.

a

b

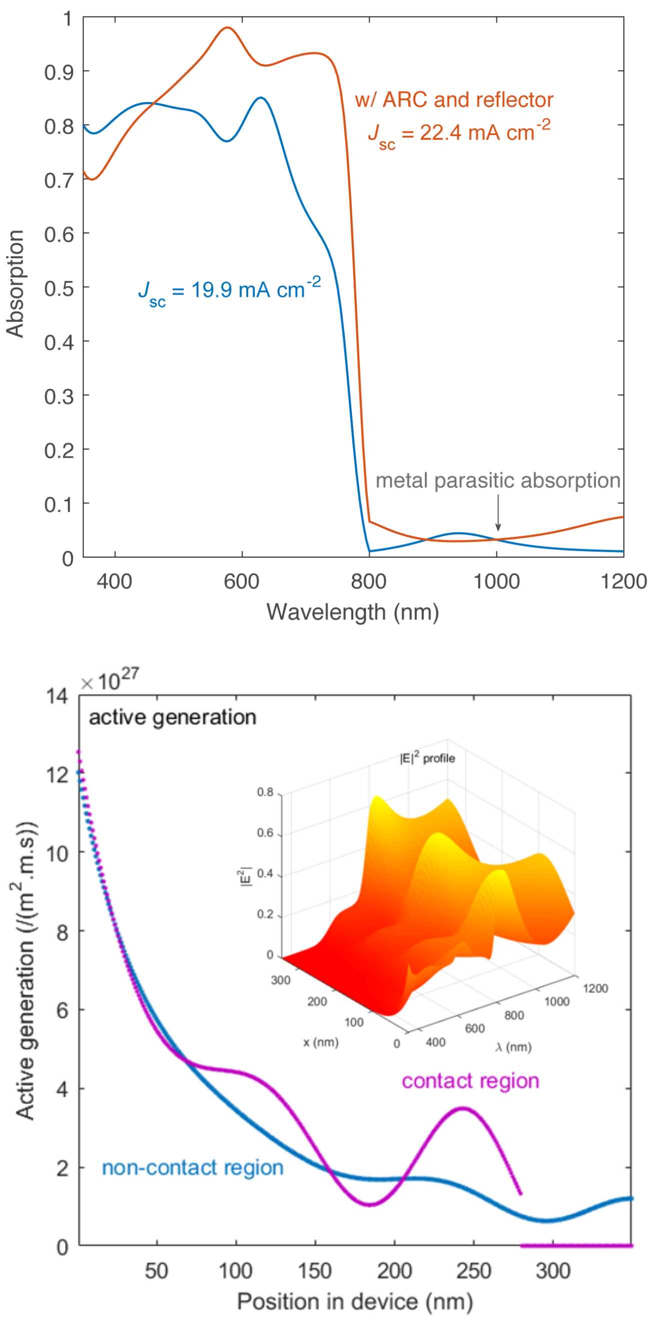

Supplementary Figure 12 | Modelling of theoretical short circuit current. (**a**) Predicted J_{SC} of 19.9 mA cm^{-2} for an 'a'-$MAPbI_3$-'b' bc-PSC obtained from a transfer matrix simulation of the absorption of the device. The model includes an aluminium rear reflector under the glass substrate. The predicted absorption spectra of the cell with inclusion of an anti-reflection coating (ARC) consisting of 110 nm of PMMA, and a planar gold reflector underneath the 350 nm of perovskite absorber, yields an improved J_{SC} of 22.4 mA cm^{-2}. (**b**), Simulated active generation profile within the contact ('a'-$MAPbI_3$ and $MAPbI_3$-'b' interfaces) and non-contact regions ($MAPbI_3$-glass interface; gap region between 'a' and 'b' electrodes) of the fabricated cell consisting of perovskite layers 280 nm and 350 nm thick, respectively. The inset shows the $|E|^2$ intensity field profile of the non-contact region of the device.

a

b

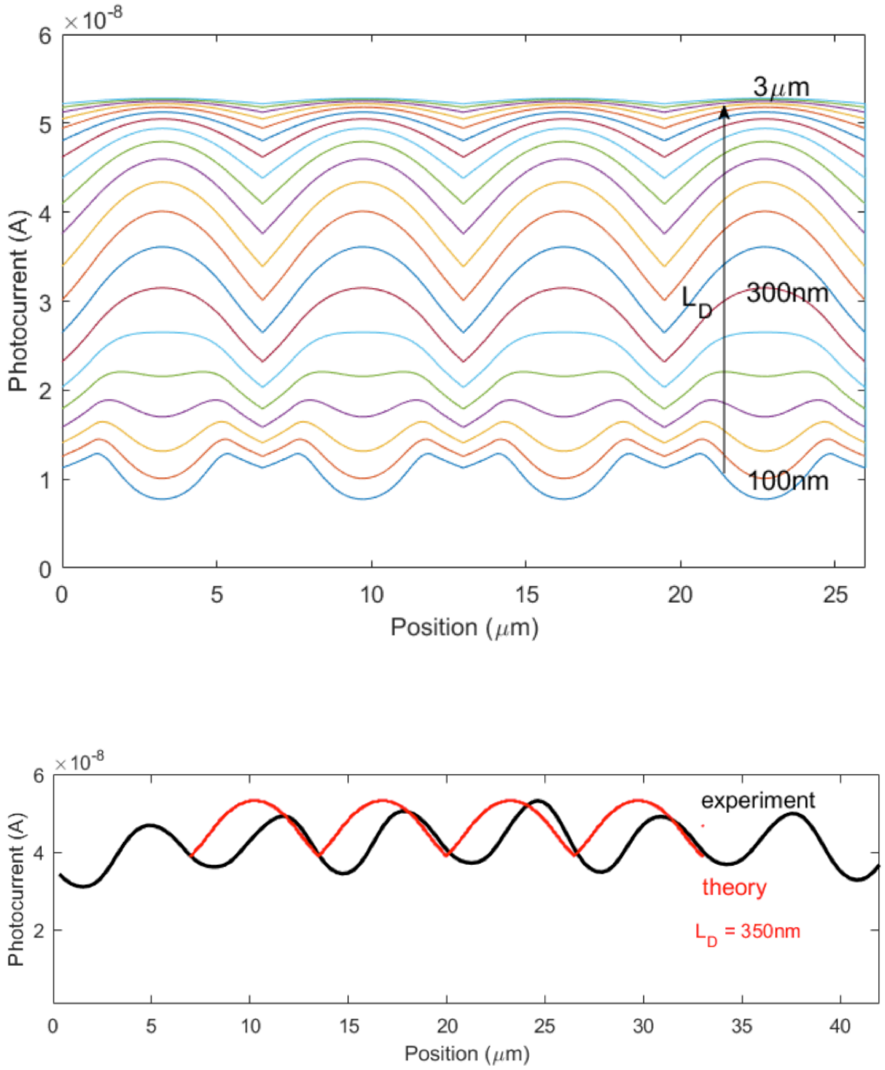

Supplementary Figure 13 | Modelling of photocurrent (PC) profile. (**a**) PC profile for different diffusion lengths, L_D, values at short-circuit condition. (**b**) Fit (red) to measured (black) PC profile under short-circuit conditions.

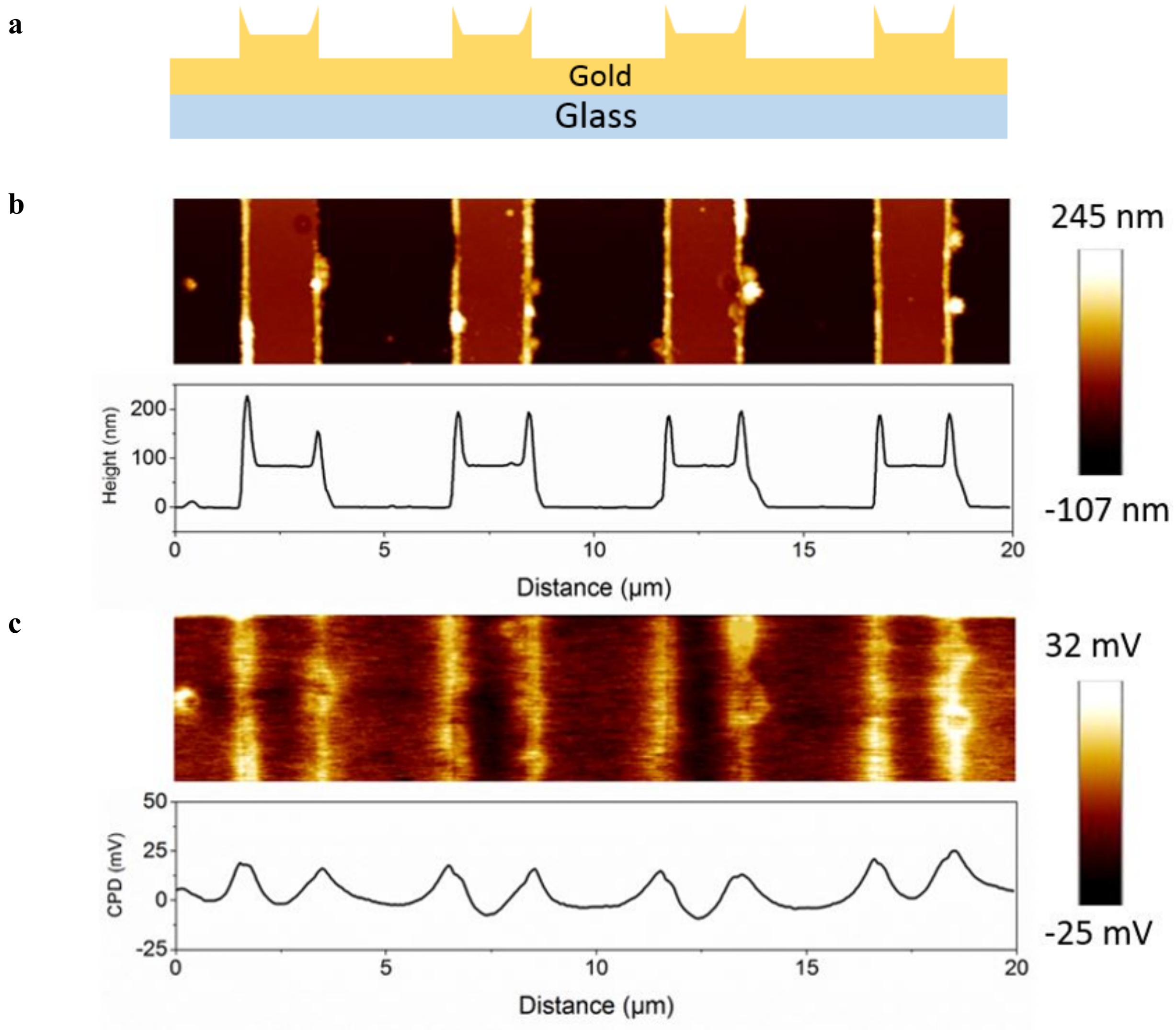

Supplementary Figure 14 | Effect of sample topography on KPFM measurement. (**a**) Schematic sketch of the test sample. (**b**) Image of an AFM topography scan. **(c)** Corresponding KPFM measurement. The topography affects the KPFM measurement in a range of around ± 15 mV only at the edge of the electrodes, and has no effect at the centre of the electrodes.

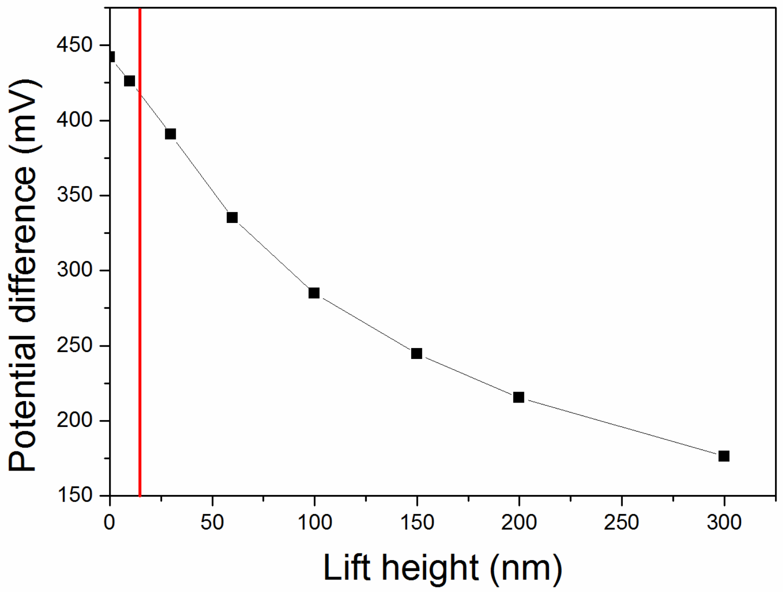

Supplementary Figure 15 | Effect of lift height of AFM tip on KPFM measurements. For KPFM images showed in the manuscript, the measurements were carried out with a lift height of 15 nm (indicated by the red line).

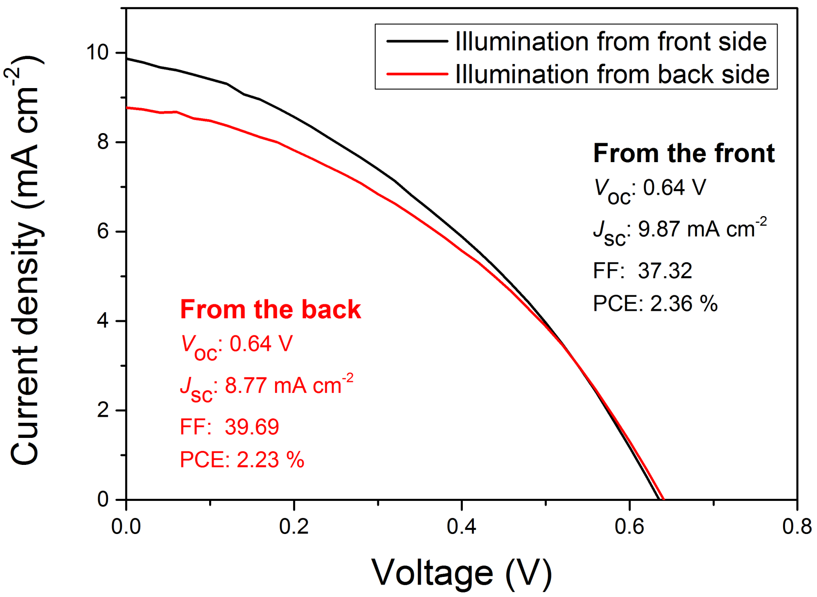

Figure 16 | Device performance with light illumination from the front (perovskite side) and back (glass side). Scanning direction: from 0 V to V_{OC}.

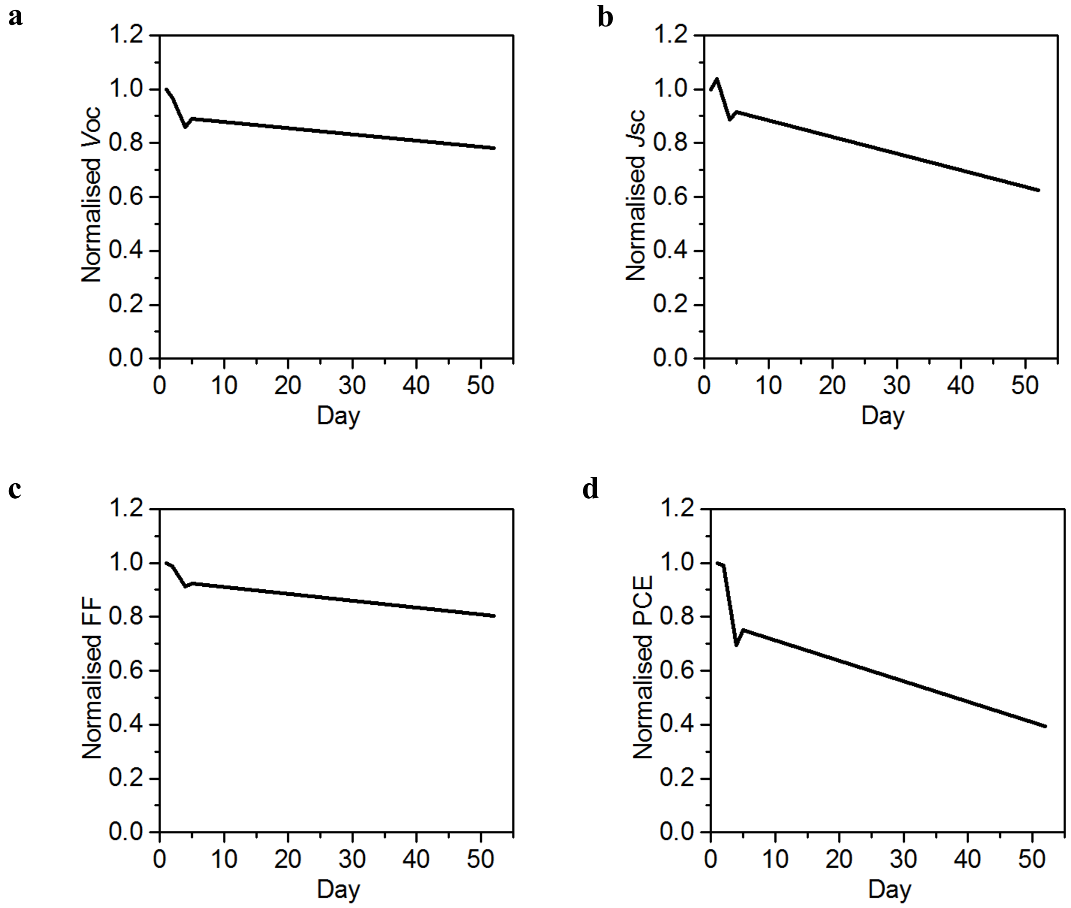

Supplementary Figure 17 | Device stability over 52 days. Time dependences of (**a**) normalized open circuit voltage (*V*oc); (**b**) normalized short circuit current density (*J*sc); (**c**) normalized fill factor (*FF*); (**d**) normalised power conversion efficiency (PCE) for an ‘a’-$MAPbI_3$-‘b’ device. The device was stored unencapsulated in an inert atmosphere in a glovebox. The initial photovoltaic values were: *V*oc = 0.64 V; *J*sc = 9.87 mA cm^{-2}; *FF* = 37.3; and PCE = 2.36 %.

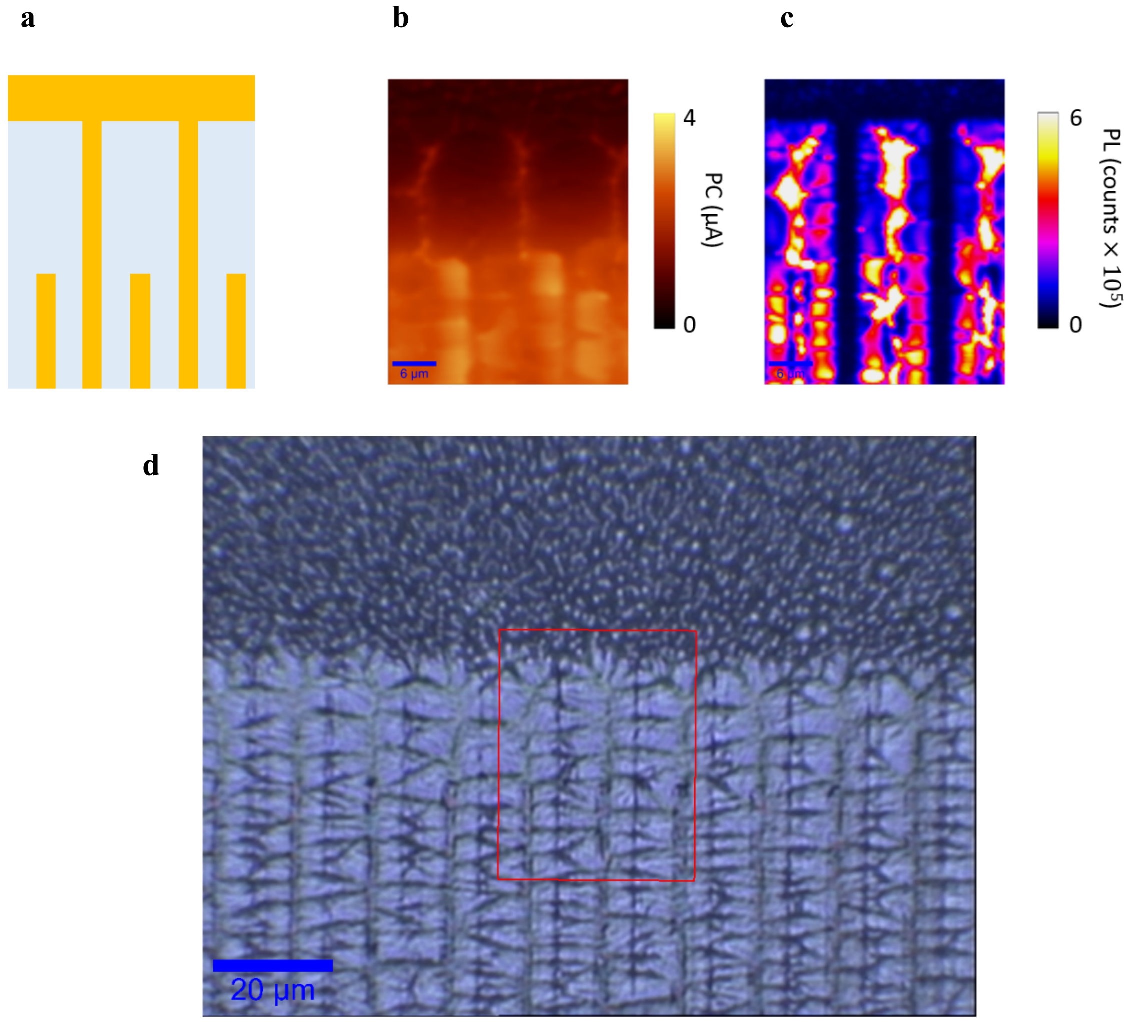

Supplementary Figure 18 | Photocurrent (PC) and photoluminescence (PL) mapping at the edge of an IDE. (**a**) Schematic of the edge of the IDE. (**b**) PC mapping. (**c**) PL mapping. (**d**) Optical image of the mapped area (red square). Photocurrent decays dramatically outside the interdigitated area.
